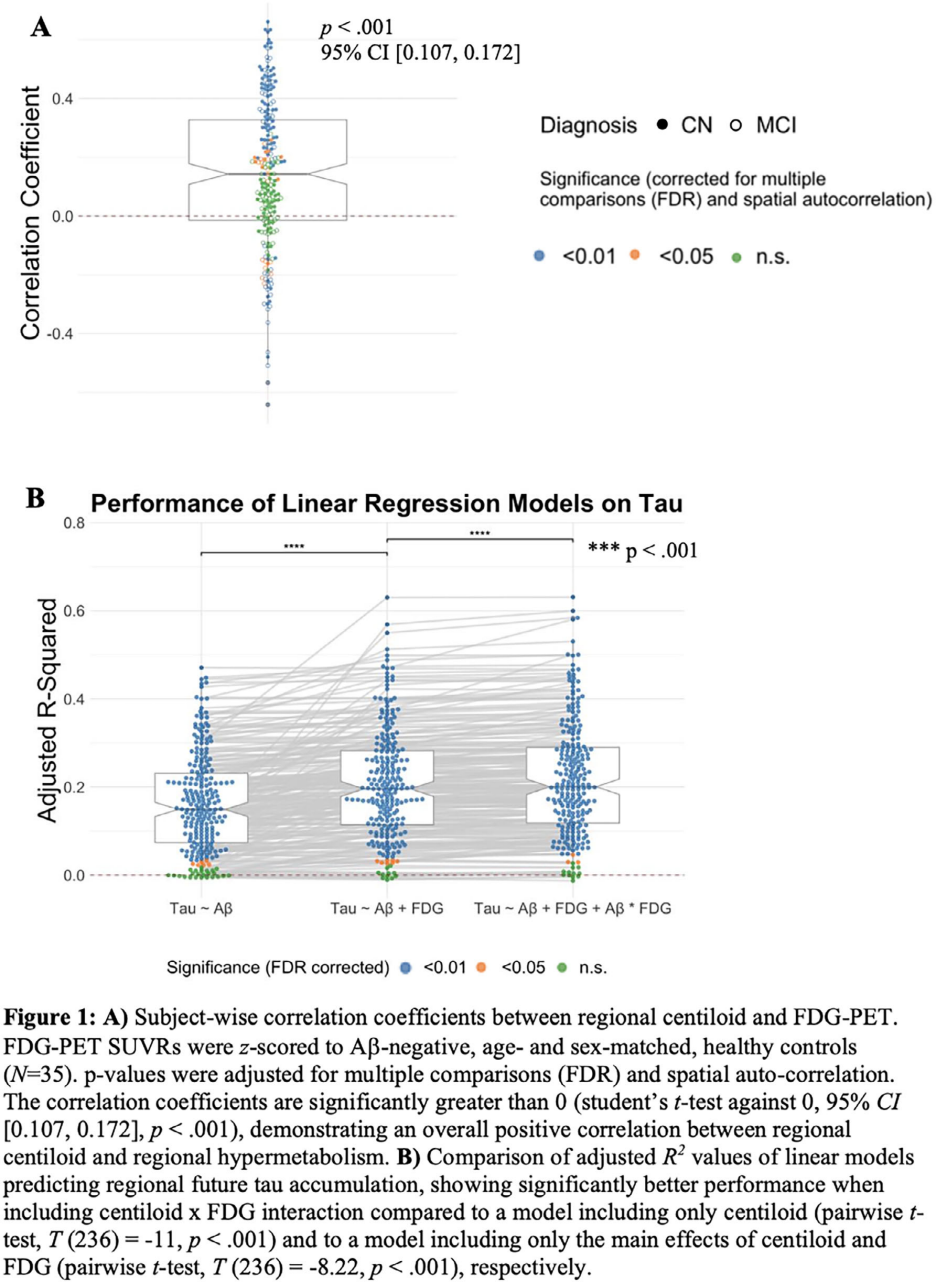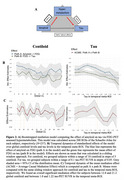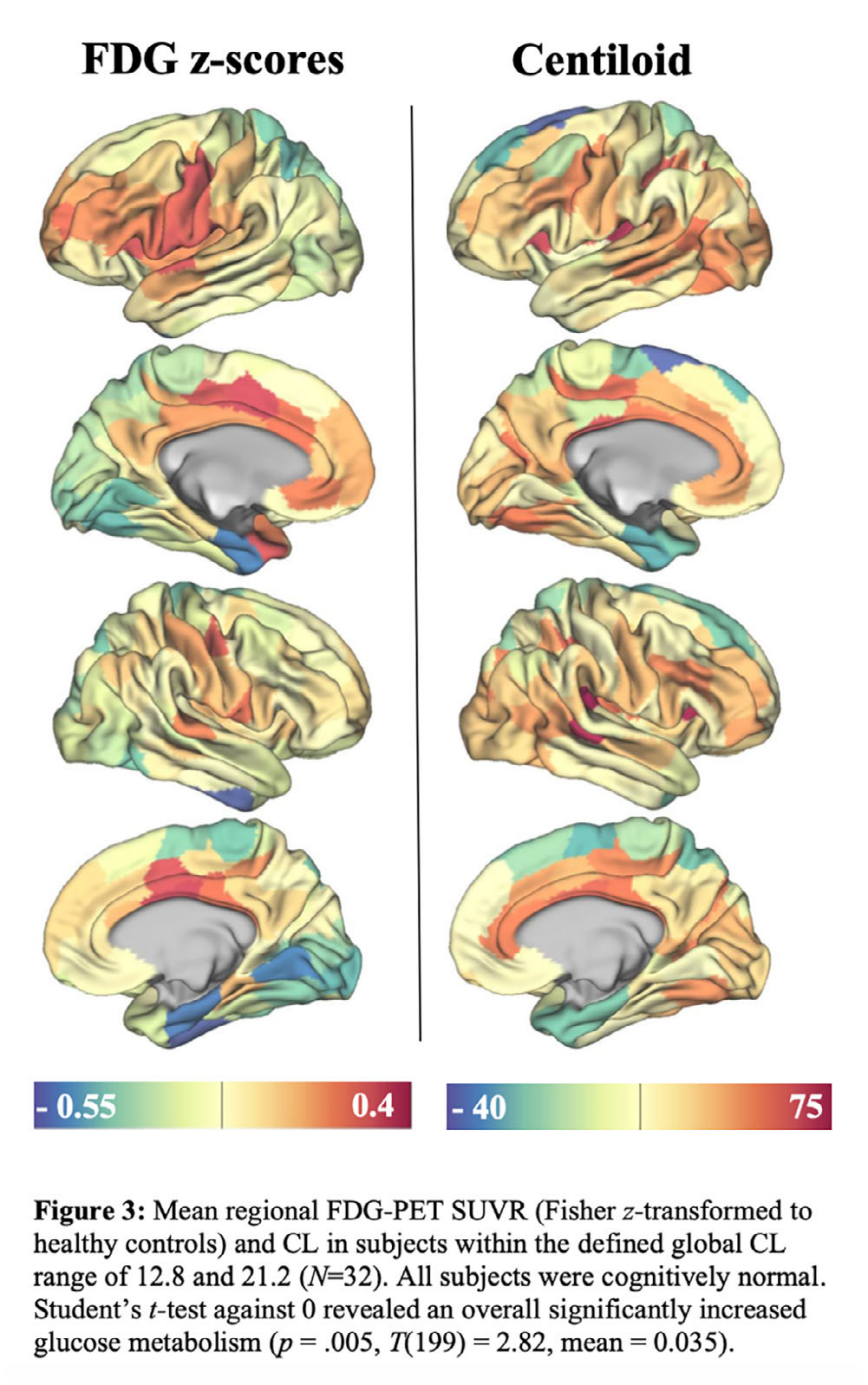# Amyloid‐induced cerebral hypermetabolism drives tau accumulation in early stages of Alzheimer’s Disease

**DOI:** 10.1002/alz70861_108551

**Published:** 2025-12-23

**Authors:** Madleen Klonowksi, Fabian Hirsch, Zeyu Zhu, Anna Steward, Anna Dewenter, Amir Dehsarvi, Lukas Frontzkowski, Carla Palleis, Davina Biel, Matthias Brendel, Nicolai Franzmeier, Sebastian Roemer‐Cassiano

**Affiliations:** ^1^ Institute for Stroke and Dementia Research (ISD), University Hospital, LMU Munich, Munich Germany; ^2^ Institute for Stroke and Dementia Research (ISD), University Hospital, LMU Munich, Munich, Bavaria Germany; ^3^ Department of Nuclear Medicine, University Hospital, LMU Munich, Munich, Bavaria Germany; ^4^ Department of Neurology, Klinikum der Ludwig‐Maximilians Universität München, Munich, Bavaria Germany; ^5^ Munich Cluster for Systems Neurology (SyNergy), Munich Germany; ^6^ University Hospital, LMU Munich, Munich Germany; ^7^ Institute for Stroke and Dementia Research (ISD), University Hospital, LMU, Munich, Bavaria Germany; ^8^ University Hospital, Ludwig‐Maximilians‐Universität, Munich Germany; ^9^ Munich Cluster for Systems Neurology (SyNergy), Munich, Bavaria Germany; ^10^ University of Gothenburg, The Sahlgrenska Academy, Institute of Neuroscience and Physiology, Psychiatry and Neurochemistry, Gothenburg Sweden; ^11^ Institute for Stroke and Dementia Research (ISD), LMU University Hospital, LMU Munich, Munich Germany; ^12^ Department of Neurology, University Hospital, LMU Munich, Munich, Bavaria Germany; ^13^ Max Planck School of Cognition, Leipzig, Sachsen Germany

## Abstract

**Background:**

The link between amyloidosis and tauopathy in Alzheimer’s Disease (AD) remains unclear. Both in‐vitro and in‐vivo studies have shown that amyloid‐beta (Aβ) induces neuronal hyperexcitability and since tau spreads trans‐synaptically in an activity‐dependent manner, Aβ‐related hyperactivity may promote tau propagation and serve as a treatment target to slow disease progression. However, the temporal dynamics of this mechanism across the disease course remains uncharacterized — an essential step for informing mechanistic models and interventions. We previously demonstrated that regional Aβ is associated with hyperconnectivity from tau epicentres to posterior regions in preclinical AD. Thus, we hypothesized that Aβ‐induced hyperexcitability and associated hypermetabolism may promote early‐stage tau accumulation and spread.

**Methods:**

From ADNI, we included subjects across the early AD continuum with baseline amyloid‐PET and FDG‐PET as a marker for neuronal activity and tau‐PET ∼4.8 years later (*N*=237). Regional FDG‐PET SUVRs were *z*‐score‐transformed relative to age‐ and sex‐matched Aβ‐negative controls (centiloid < 0). We computed within‐subject correlations of regional centiloid and FDG‐PET. Subject‐specific mediation models were used to test whether higher Aβ promotes later tau accumulation via FDG‐PET increases. We employed a sliding‐window approach to quantify the mean mediation effect across different centiloid and tau levels.

**Results:**

Higher regional centiloid is significantly associated with stronger regional metabolism (mean *r*=0.14, *p*<0.001). Further, future tau‐PET is best predicted by linear models including Aβ and FDG and their interaction term (Figure 1). We found an overall significant mediation effect of baseline Aβ through FDG‐PET on tau‐PET ∼4.8 years later (ACME=0.013, *p*<0.001, *T*(236)=3.75). Assessing the temporal dynamics of the mediation effect throughout the disease course showed a significant positive mediation pathway between 12.8‐21.2 centiloids and 1.0‐1.22 tau‐PET SUVR in the temporal meta‐ROI (Figure 2). Subjects within this centiloid range show significantly increased FDG‐PET metabolism, promoting downstream tau‐PET increases (Figure 3). As expected, the FDG‐tau association decreases as the disease progresses, attenuating the mediation effect.

**Conclusion:**

Our results suggest that early‐stage Aβ‐related hypermetabolism drives tau accumulation already at low centiloid levels, preceding tau positivity in the temporal meta‐ROI and decreases as disease progresses. Trials targeting neuronal hyperactivity may be most effective at early‐stage Aβ deposition and low fibrillar tau.